# Biological interaction of living cells with COSAN-based synthetic vesicles

**DOI:** 10.1038/srep07804

**Published:** 2015-01-15

**Authors:** Màrius Tarrés, Elisabetta Canetta, Eleanor Paul, Jordan Forbes, Karima Azzouni, Clara Viñas, Francesc Teixidor, Adrian J. Harwood

**Affiliations:** 1 Institut de Ciència de Materials de Barcelona (ICMAB-CSIC), Campus UAB, E-08193, Bellaterra, Spain; 2 School of Biosciences, Cardiff University, Museum Ave, Cardiff CF10 3AX, United Kingdom

## Abstract

Cobaltabisdicarbollide (COSAN) [3,3′-Co(1,2-C_2_B_9_H_11_)_2_]^−^, is a complex boron-based anion that has the unusual property of self-assembly into membranes and vesicles. These membranes have similar dimensions to biological membranes found in cells, and previously COSAN has been shown to pass through synthetic lipid membranes and those of living cells without causing breakdown of membrane barrier properties. Here, we investigate the interaction of this inorganic membrane system with living cells. We show that COSAN has no immediate effect on cell viability, and cells fully recover when COSAN is removed following exposure for hours to days. COSAN elicits a range of cell biological effects, including altered cell morphology, inhibition of cell growth and, in some cases, apoptosis. These observations reveal a new biology at the interface between inorganic, synthetic COSAN membranes and naturally occurring biological membranes.

All biological membranes are formed by self-assembly of lipid molecules, with their polar head groups exposed to the aqueous environment and their fatty acid tails orientated internally, to create a hydrophobic barrier^1^. These membranes establish the boundary between the living cells and the external environment, and form barriers between internal cell compartments and organelles. Control of biomolecular flow across, and within, membranes is an important element for cell regulatory processes, and this flux is achieved by transport and channel proteins, or re-sculpting membranes via vesicle intermediates. A diversity of lipid membrane components increase complexity by imparting different physicochemical membrane properties, such as membrane thickness, lateral diffusion rate and curvature^2^,^3^. Membrane integrity is essential for control of the distribution of ions, biomolecules and metabolites, and when disrupted leads to a rapid loss of cell viability.

Cobaltabisdicarbollide, [3,3′-Co(1,2-C_2_B_9_H_11_)_2_]^−^, commonly known as COSAN, is a complex, boron-based anion that forms vesicles and membranes in aqueous solution^4^. COSAN comprises of a central cobalt atom sandwiched between two carboranyl clusters^5^,^6^,^7^ (Figure 1A). Unlike the polarized lipid molecules that form biological membranes, COSAN has a dispersed net negative charge^8^ but a hydrophobic surface, and forms intermolecular interactions via its weakly polarized B-H and C-H bonds^9^,^10^,^11^. The simultaneous hydrophobic and hydrophilic nature of COSAN makes it soluble in both water and oils, and at a critical concentration it forms small, monolayer vesicles, and eventually micelles, with dimensions only slightly smaller than those formed from lipid bilayers^4^,^12^.

We have previously shown that COSAN vesicles are able to pass directly through synthetic lipid membranes with zero-order kinetics^12^. We further showed that when applied to cells growing in culture, COSAN passed into cells without disrupting membrane integrity^13^. Here, we examine the effects of COSAN and some of its derivatives on cell behaviour, demonstrating that COSAN has a range of biological effects and in particular causes cell growth arrest in all cell types investigated. These effects are reversible, suggesting a biological interaction rather than chemical toxicity.

## Results

### COSAN interactions with living cells

**Figure 1:**
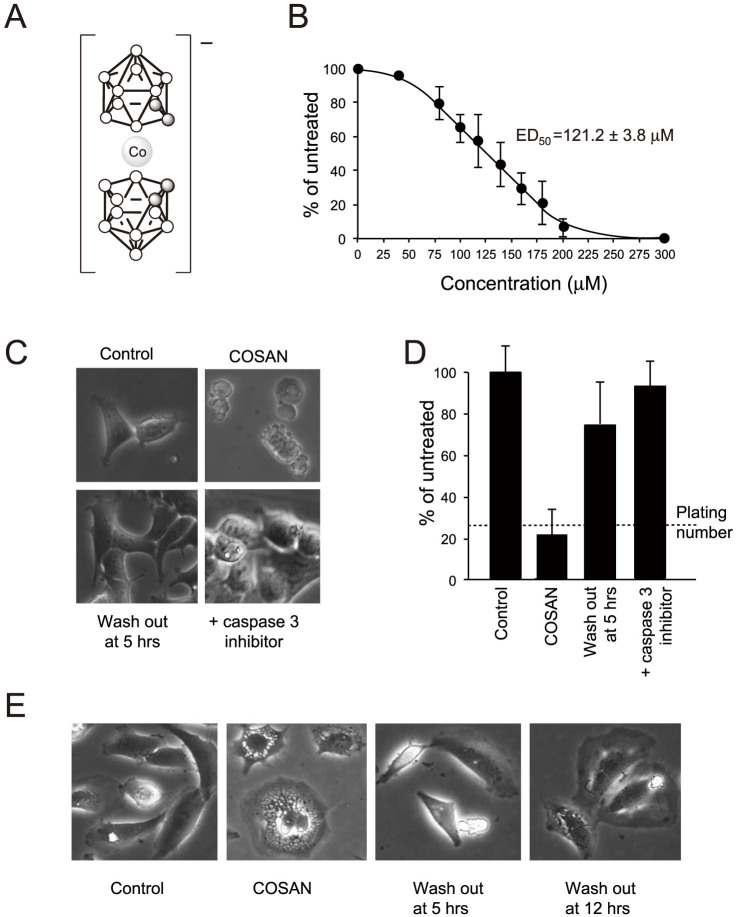
The effect of COSAN on mammalian cells. (A) Structure of COSAN. White spheres = boron atoms, Grey spheres = carbon atoms (B) Proliferating HEK293 cells were plated in the presence of increasing COSAN concentrations and cells counted after 4 days, expressed as percentage of growth of untreated cells. Experiments were carried out in triplicate, and plotted as mean ± SD. ED_50_ values were established for three independent experiments using exponential curve fitting, and used to calculate mean ± SD ED_50_ values. (C) Phase contrast images of cells grown in the absence (Control) or presence (COSAN) of 200 μM COSAN for 24 hours. Second panel: COSAN treated HEK293 cells show a blebbing morphology often associated with apoptotic cells. Third panel (Wash out at 5 hrs): cells grown in the presence of 200 μM COSAN for 5 hours, before the cells were washed and grown in COSAN-free medium for a further 19 hours. Fourth panel (+caspase 3 inhibitor): cells plated for 24 hours in COSAN and 100 nM Ivachtin (a caspase 3 inhibitor). (D) Final cell density of HEK293 cells grown for 48 hours in the absence (Control); presence of 200 μM COSAN (COSAN); (Wash out at 5 hrs) following removal after 5 hours and further growth for 43 hours in fresh medium; (+caspase 3 inhibitor) or with COSAN plus caspase 3 inhibitor (100 nM Ivachtin) for 24 hours, followed by wash out and further growth for 24 hours in fresh medium, without COSAN or caspase 3 inhibitor. Data is plotted as percentage of untreated cell cultures, mean ± SD for 3 independent experiments. (E) HeLa cells show an unusual, highly vacuolated morphology within the perinuclear cytoplasm when grown in COSAN for 24 hours. Following wash out of COSAN at either 5 or 12 hours the cells revert to the morphology seen in untreated cells (control).

We examined the effect of COSAN on a range of mammalian cell lines: HEK293, HeLa, 3T3 cells, growing as adherent monolayers, and a lymphoblastoid line THP-1, growing in suspension. In all concentrations examined, COSAN treatment had no immediate effect on cell viability, with cells showing no signs of membrane disruption. For most cells and culture conditions, we observed cell rounding within minutes to an hour of addition of COSAN, but with adherent cells remaining attached to the substratum. Cell rounding was also seen with cells plated in PBS, similar aqueous conditions to those used in previous biophysical studies in which COSAN forms vesicles^12^. In addition to morphological change, all cells tested arrested proliferation and growth. Using cell proliferation in 4 day culture as an assay, we calculated ED_50_ values of between 99–157 μM (Figure 1B, Table 1).

For some cells and growth conditions, however we observed additional effects. When HEK293 cells were plated at low density, and hence had a higher proliferation rate, they began to bleb after 8 hours of treatment (Figure 1C), having the appearance of cells entering apoptosis^14^ with large numbers of cells dying at 24 hours. If HEK293 cells grown under these conditions were treated with both COSAN and the caspase-3 inhibitor Ivachtin to block apoptosis, cells remained viable for 24 hours (Figure 1C), and fully recovered if COSAN was then washed out and cells grown for a further 24 hours (Figure 1D). Finally, if HEK293 cells grown at low density were treated with COSAN for 5 hours, 3 hours before obvious apoptosis, and then COSAN was washed out, cells recovered and began to grow and proliferate normally (Figure 1C and 1D).

In contrast, HeLa cells grown under the same conditions showed no evidence for blebbing or cell death. Instead, they took on an unusual, highly vacuolated appearance after 24 hours (Figure 1E). When COSAN was applied to HeLa cells for 5 hours and then removed, cells reverted to the same morphology as untreated cells (Figure 1D), indicating again that COSAN-mediated effects were reversible. Even when cells were exposed to COSAN for 12 hours and then washed free for a further 12 hours, the majority of cell showed no vacuolation, and those cells with vacuoles showed only minimal effects compared to 24 hour exposure. We conclude that of the cells examined apoptosis is restricted to HEK293 cells, and other cell types show different cellular changes not associated with cell death. As these additional effects require long-term exposure, greater than 5 hours, and are reversible if COSAN is removed before this time, we conclude that COSAN is not cytotoxic in the short term.

**Figure 2:**
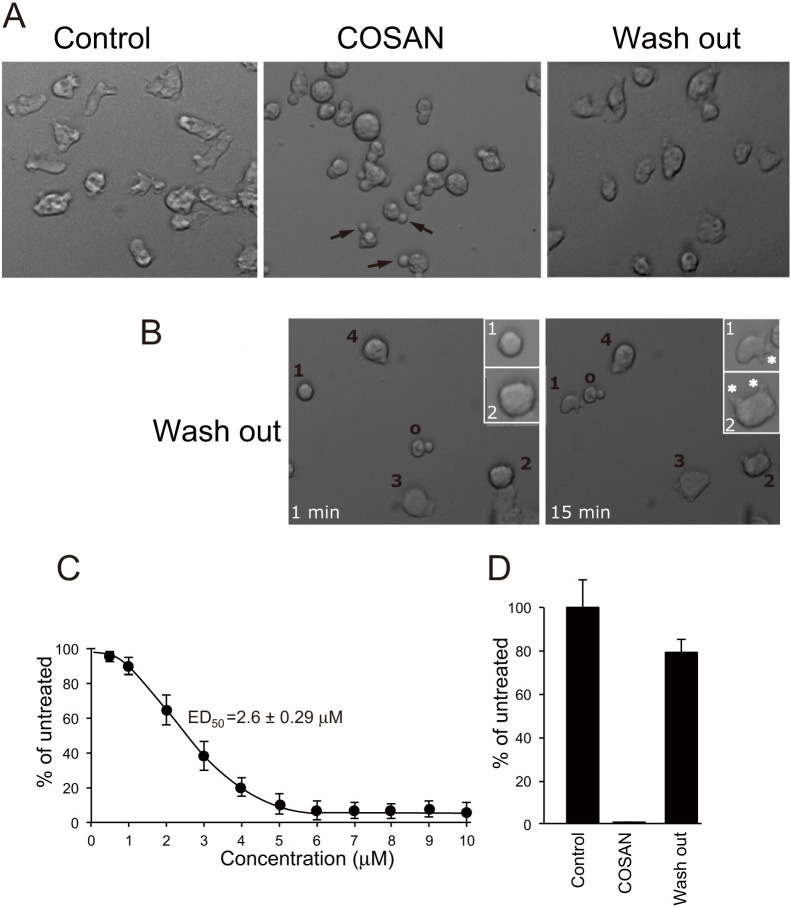
The effect of COSAN on *Dictyostelium* cells. (A) *Dictyostelium discoideum* cells treated with 10 μM COSAN. Cells were photographed prior to COSAN addition (Control), 30 min after COSAN addition (COSAN) and 20 hours after COSAN removal (Wash out). Arrows indicate the cellular protrusions seen for most COSAN treated cells (B) Cells were observed by videomicroscopy following COSAN wash out. Images show the same cells immediately (1 min) and 15 minutes following COSAN wash out. Cells 1–4 indicate cells attached to the substratum, and beginning to show motility. Cell “o” has not yet recovered from COSAN treatment and is still unattached. Cells 1 and 2 are inset at a higher magnification to show the emergence of pseudopods (marked *) 15 min after COSAN removal. (C) *Dictyostelium* cells were plated in COSAN concentrations up to 10 μM and cells counted after 4 days. Cell number at 4 days as the percentage of untreated cells is plotted against COSAN concentration, and ED_50_ is derived from 3 independent experiments, and shown as the mean ± SD value. (D) Final cell density of *Dictyostelium* cells grown and counted after 6 days in the presence (COSAN) or absence (Control) of 500 μM COSAN. At this point, the COSAN treated cells were washed and grown in COSAN-free medium and grown for a further 6 days (wash out). Histogram shows mean ± SD of 3 independent experiments as percentage of the mean untreated cell cultures. Control and COSAN samples are based on counts at 6 days, Wash out was counted at 12 day (6 days with COSAN, followed by 6 days without).

To probe the effect of COSAN on cell growth without these longer-term confounding effects, we switched the investigation to *Dictyostelium* *discoideum* cells, a eukaryotic cell that is evolutionarily close to animal cells, but lacks caspase-mediated apoptosis^15^,^16^. We found that these cells were much more sensitive to COSAN, with an ED_50_ of 2.6 ± 0.29 μM, and total arrest of proliferation occurring at 4 μM (Figure 2A–C). Under phase contrast microscopy, treated cells retained the refractile appearance of living cells, but rounded up and often exhibited a single large protrusion (Figure 2A). Consistent with a lack of cytotoxicity, cells rapidly reverted to the untreated state following COSAN removal (Figure 2A and 2B). Due to the morphological change, cells became loosely attached to the underlying substratum. Cell detachment however cannot explain the growth arrest, as untreated *Dictyostelium* cells grow well in shaking suspension (Figure 2C). As seen for mammalian cells, cell rounding was also observed when COSAN was added in phosphate buffer (KK2) or water. In growth medium *Dictyostelium* cells remained viable for long periods in COSAN. To date, we have found no limits for *Dictyostelium*, with cultures remaining viable for more than 1 month (37 days) in 10 μM COSAN, and recovering after removal. In addition, cells were viable and grew well in COSAN-free culture, even after prolonged exposure to 500 μM of COSAN, almost 200 times the ED_50_ value (Figure 2D).

### Modification of COSAN alters its cellular potency

**Figure 3:**
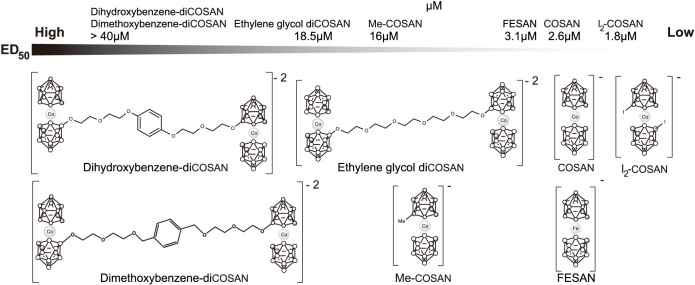
A chemical series based on COSAN reveals increased potency of I_2_-COSAN on living cells. (A) A schematic view of the structure of the metallabisdicarbollides investigated in this study. The ED_50_ values for *Dictyostelium* are given for each compound, ranked from least to most potent.

**Figure 4:**
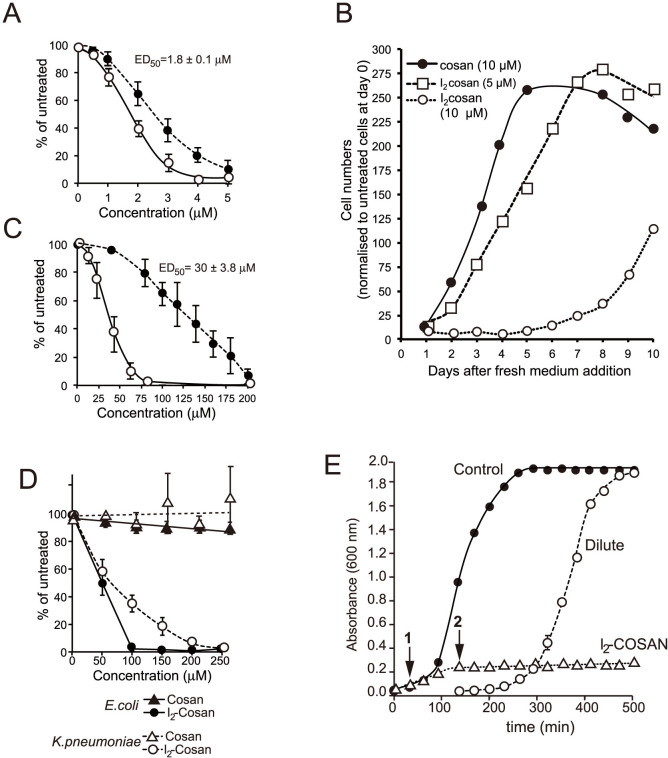
Analysis of I_2_-COSAN on cell growth. (A) *Dictyostelium discoideum* cells were plated in I_2_-COSAN concentrations up to 10 μM and cells counted after 4 days. Cell number at 4 days as the percentage of untreated cells is plotted against COSAN concentration, and ED_50_ (mean ± SD) for I_2_-COSAN is shown on graph. The curve for COSAN is repeated from Figure 2A for comparison (dashed line). (B) *Dictyostelium* cells were cultured in shaking suspension in the presence of COSAN and I_2_-COSAN at the concentrations indicated (5 or 10 μM) for 6 days, then washed and re-suspended in COSAN-free medium. Growth was then monitored by cell counting over a 10 days period. (C) HEK293 cells were plated in increasing I_2_-COSAN concentrations and effect on growth measured after 4 days, expressed as percentage of growth of untreated cells. The ED_50_ (mean ± SD) for I_2_-COSAN is shown on graph. The curve for COSAN is repeated from Figure 1B for comparison (dashed line). (D) Dose curves for the effect of COSAN and I_2_-COSAN on the bacterial species *E. coli* (strain B/r) and *Klebsiella pneumonia*. The effect on growth was monitored at 240 min and is plotted as the percentage of the untreated control. (E) Growth of *E. coli* B/r was monitored by Absorbance at 600 nm in the presence or absence of 100 μM I_2_-COSAN. I_2_-COSAN was added at 30 minutes of growth (arrow 1). After 105 minutes, the concentration of I_2_-COSAN was lowered to 25 μM by dilution (arrow 2) and the growth of the culture monitored for a further 395 min (curve marked as Dilute).

Using the more sensitive *Dictyostelium* cells, we tested a range of chemical variations and modifications of COSAN (Figure 3). Substitution of cobalt for iron as the central metal (FESAN) made no major difference to compound potency. However, methylation of COSAN, or linking two COSAN clusters via a PEG chain decreased cell potency (Figure 3). In contrast, addition of iodine to make di-iodo-COSAN (I_2_-COSAN) increased potency (Table 1; Figure 3 and 4A). *Dictyostelium* cell recovery after I_2_-COSAN treatment took longer than COSAN and was dose-dependent, suggesting a stronger interaction with its cellular targets (Figure 4B). When tested on mammalian cell cultures, again I_2_-COSAN was more potent than COSAN (Figure 4C). In addition, we tested the influence of different biocompatible cations: salts of COSAN, Li^+^ and Na^+^ (ED_50_ values were 2.5 and 2.6 μM, respectively); salts of FESAN, Li^+^ and Na^+^ (ED_50_ values were 3.2 and 3.1 μM, respectively); salts of I_2_-COSAN, H^+^ and Na^+^ (ED_50_ values of both salts was 1.8 μM), but found no differences in effects on cells. On this basis, all biological tests were carried out with Na^+^ as the cation of the COSAN anion and its derivatives.

We had found no, or very little, effect of COSAN on the growth of bacteria. In contrast, I_2_-COSAN arrested growth of the two bacterial species we tested, *E. coli* and *K. pneumoniae*, (Figure 4D). Interestingly, we found that *E. coli* was significantly more sensitive to I_2_-COSAN than *K. pneumoniae*. Growth arrest was reversed by I_2_-COSAN removal or dilution to below its ED_50_ value (Figure 4E).

### Direct visualization of I_2_-COSAN within cells

**Figure 5:**
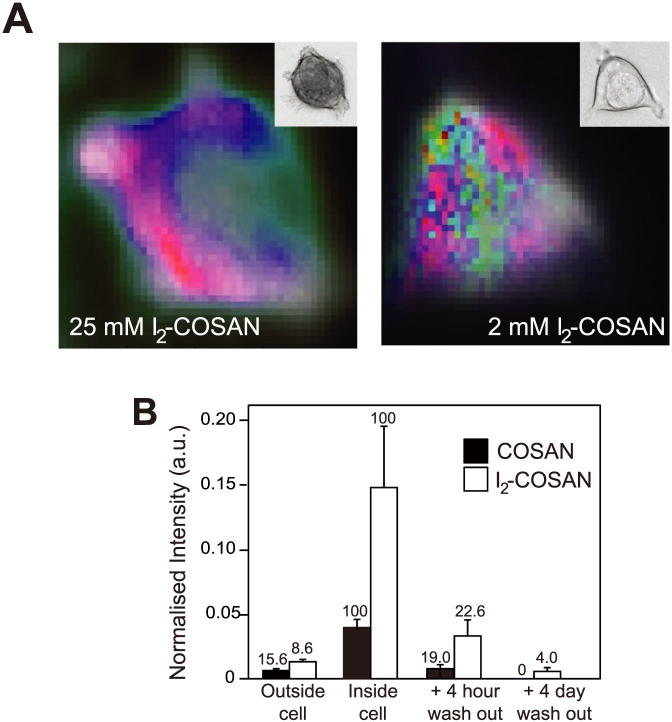
Monitoring COSAN and I_2_-COSAN in living cells. (A) Cellular imaging of HEK293 cells treated with 25 and 2 mM I_2_-COSAN. Images show phase contrast image (inset) and Raman chemical images at 2570 cm^−1^, the peak signal for the B-H bond. I_2_-COSAN accumulation is shown on a red scale, ranging from red/pink colour corresponding to high I_2_-COSAN concentration to white for lowest concentration. Green marks the nucleus, based on the additional spectra characteristic of nuclear material, and blue marks the cell membrane. Images were collected with confocal optics and represent a single optical plane. (B) A histogram showing the mean ± SD intensity of the B-H reference peak for COSAN and I_2_-COSAN both inside and outside cells (n = 20 cells) after 30 minute incubation; and following wash out in fresh PBS for 4 hours and 4 days once the compounds have been removed, the cells washed and then replaced in fresh medium. Percentage of mean maximum value is shown above each bar.

To investigate why I_2_-COSAN was more potent than the original COSAN molecule, we used micro-Raman spectroscopy to compare uptake and release of both molecules. As previously reported, the distinctive vibrational peak at 2570 cm^−1^ of B-H bonds provides a means of direct visualization and quantification of COSAN and I_2_-COSAN in living HEK293 cells^13^, showing that both COSAN and I_2_-COSAN accumulates inside cells (Figure 5). In fact, we found that both COSAN and I_2_-COSAN reach intracellular concentrations substantially greater than in the external medium (PBS). This is illustrated well in the case of I_2_-COSAN, where an extracellular 2 mM concentration of I_2_-COSAN, a concentration too low for normal detection, accumulated in 30 minutes to a concentration of approximately 25 mM within cells, a greater than 10-fold rise (Figure 5A, supplementary Figure. S1 online). Although both molecules possessed the ability to accumulate against the concentration gradient, we observed that I_2_-COSAN reached an intra-cellular concentration 3–4 times that of COSAN (Figure 5B). When COSAN or I_2_-COSAN was then removed from the medium, they initially remained within the cell and then were gradually lost over the preceding hours. However 4 hours after COSAN or I_2_-COSAN removal from the external environment, there was more retained inside the cell than originally applied to the outside, and I_2_-COSAN was still detectable within cells after 4 days (Figure 5B). These observations indicate that the rate of entry of both COSAN and I_2_-COSAN is greater than the exit rate, and can explain the intracellular accumulation.

Although absolute concentration of I_2_-COSAN inside the cells was higher than COSAN, in both cases the concentration 4 hours after removal was 20% of the total peak concentration (Figure 5B). This indicated that COSAN and I_2_-COSAN exited cells at the same rate, and did not explain why intracellular I_2_-COSAN was higher than COSAN. To investigate this further, we measured the octanol-water Partition Coefficient (P) as an indication of lipophilicity (supplementary Figure. S2 online), and found that I_2_-COSAN has a partition value (P = 151.0) approximately 3.5-fold higher than COSAN (P = 43.7). This study indicates that I_2_-COSAN possesses significantly higher lipophilicity than COSAN.

The ability to accumulate and measure I_2_-COSAN within cells that are incubated in I_2_-COSAN concentrations below the detection threshold offers a strong signal contrast with which to image the subcellular distribution. As previously reported for treatment with higher COSAN concentrations, cells treated with 2 mM I_2_-COSAN showed that although present within both the cytoplasm and nucleus, I_2_-COSAN was not homogeneously distributed, with some cytoplasmic regions showing higher concentrations than others (Figure 5A). Currently, we do not have sufficient spatial resolution to correlate these higher concentration regions with known sub-cellular structures. It is very clear, however, that there was no substantial accumulation in the plasma membrane, consistent with our previous biophysical observations that COSAN passed directly through artificial membranes^12^.

## Discussion

The interaction of COSAN and related molecules with synthetic and biological membranes was described in previous reports^12^,^13^, and here we have explored the biological interactions with living cells. COSAN and I_2_-COSAN form vesicles in aqueous solution^4^ and have the unusual property of transiting lipid bilayers with zero order kinetics^12^, where rate is independent of the initial concentration. Previously, we observed fusion and transit of COSAN vesicles through the lipid membrane of the liposomes^12^, and COSAN entry into living cells^13^. In the experiments reported here, we show that COSAN entry into cells neither disrupts the plasma membrane nor is immediately cytotoxic. This is consistent with the previous biophysical experiments^12^ that demonstrated that the boron-rich anion COSAN and its I_2_-COSAN derivative cross through cell-free artificial lipid bilayer synthetic membranes, either prokaryotic (DPhPC: 1,2-diphytanoyl-sn-glycero-3-phosphocholine) or eukaryotic (1,2-dioleoyl-sn-glycero-3-phosphocholine, DOPC).

We found that for most eukaryotic and prokaryotic cell cultures, cells remain viable with prolonged COSAN treatment, but have arrested cell growth and proliferation, indicating a cytostatic, not cytotoxic, effect. When COSAN is washed from the cells, this cytostatic effect is reversed and they resume growth. However, under some conditions, additional cell effects were observed: proliferating HEK293 cells exhibiting apoptosis and HeLa cells forming large intracellular vacuoles. These additional, cell-specific events require exposure of many hours, contrasting to the morphological changes, followed by growth arrest, that occurs rapidly after COSAN exposure in all eukaryotic cells. These observations suggest that the interaction of COSAN with cells is more than a simple physicochemical process and may entail more active interactions with intracellular cell signalling pathways that control proliferation, death or membrane dynamics.

The nature of these interactions is currently unknown. With our current imaging techniques we cannot determine whether separate COSAN vesicles form within cells as seen in artificial liposome experiments or whether COSAN forms more complex structures within the cytoplasm, such as hybrid COSAN and phospholipid membranes. We note however the association between membrane fluidity, cell proliferation arrest and apoptosis seen with other small molecules, such as methyl jasmonate^17^, and it is possible that all the biological effects we observe could arise due to membrane interactions. Alternatively, COSAN may mediate its effects due to protein interactions either by enzyme inhibition via active site binding, such the modified COSAN molecules that inhibit enzymes^18^,^19^, or more general interference by binding to protein surfaces. More detailed analysis is required to resolve these mechanistic possibilities.

The effects of COSAN are seen in a broad range of conditions, spanning different counter ions (H^+^, Li^+^ and Na^+^) and cell media and buffers. In addition, the core effects on cell morphology and cell growth arrest are conserved over many cell types, including *Dictyostelium* and bacteria, although the sensitivity to COSAN and its derivatives varies widely over different cell types. Changing the structure of the COSAN had a large effect on the potency of its effect, with diCOSAN structures having no or very little effect, whereas addition of iodine (I_2_-COSAN) enhances its biological effects.

To investigate why I_2_-COSAN has higher potency than COSAN, we used micro-Raman spectroscopy to monitor both COSAN and I_2_-COSAN within living cells. We found that both molecules exit cells at a slower, but equal, rate in comparison to entry. This leads to accumulation within the cell against the concentration gradient, however I_2_-COSAN accumulates to a higher level. Currently, we do not know whether COSAN accumulation reaches a maximum concentration, however the cell biological effects described in this paper occur at much lower concentrations of COSAN or I_2_-COSAN than used for imaging, and so there is plenty of capacity for both to accumulate to a level with significant effects.

A key difference between COSAN and I_2_-COSAN is the approximate 3.5 times higher lipophilicity of the I_2_-COSAN molecule, which suggests a higher affinity for the lipid environment. This difference is also consistent with observations on artificial membranes^12^, where the rate-limiting factor for COSAN and I_2_-COSAN translocation is partitioning between lipid and water phase, and the slower permeation rate seen for I_2_-COSAN could result from its greater affinity for lipids. Importantly, we do not see either COSAN or I_2_-COSAN accumulation within the plasma membrane of cells further demonstrating that COSAN anions do not accumulate within the lipid membrane phase.

A valuable feature of the inorganic boron-based COSAN membranes reported here is that they are not present in nature and therefore unlikely to be further modified or degraded by cellular enzymes. Consistent with this, we have observed no chemical modification of COSAN or I_2_-COSAN in the cell systems reported here (see supplementary Figure S3 online). This biologically inert feature may offer new opportunities for drug design and molecular delivery systems. Recent work demonstrates that functionalized I-COSAN vesicles show good biodistribution and can be imaged throughout whole mice^20^, and we would predict that they would not show major toxicity if concentrations are maintained below 100 μM. COSAN, and other borane clusters, can be coupled to bioactive molecules, such as inhibitors, siRNA and protein ligands^21^,^22^,^23^,^24^, and may have the potential to carry these molecules across the plasma membrane and into cells. COSAN vesicles could also be used to encapsulate water soluble or hydrophilic compounds, however it is currently unclear how the topology of the COSAN vesicles changes as they merge with lipid bilayer membranes and whether this would deliver into the cell or disperse on the cell surface.

As COSAN can accumulate to high concentrations in living cells, this may enhance potential therapeutic effects. For example, its cell accumulation may be beneficial for Boron neutron capture therapy (BNCT) proposed for cancer therapy^25^,^26^,^27^, or other clinical usages. We also note the differential sensitivity between cell state and cell species. In tissue culture, we observed cell death in proliferating cells following prolonged COSAN treatment (Figure 1C), however confluent monolayers appeared unaffected. If this is translated to the *in vivo* context, then it may be possible to target rapidly growing cells. *Dictyostelium* amoebae show a much higher sensitivity to COSAN and its derivatives. If this higher sensitivity to COSAN were also present in other unicellular eukaryotes, it may offer an amoebicidal agent to target protozoan and amoebozoan pathogens.

Placed in the context of earlier papers, these observations demonstrate that COSAN is able to accumulate within cells without affecting membrane integrity. Once inside the cell, COSAN is not immediately cytotoxic, but is cytostatic over the long term, and cells recover following its removal. Specific biological effects, however, vary with cell type and conditions. These results reveal novel biology interactions at the interface of biological and synthetic membranes.

## Methods

### COSAN and its derivatives

Single COSAN moieties Cs[3,3′-Co(1,2-C_2_B_9_H_11_)_2_], Cs[COSAN]^6^; Cs[3,3′-Fe(1,2-C_2_B_9_H_11_)_2_], Cs[FESAN]^6^; Cs[3,3′-Co(8-Me-1,2-C_2_B_9_H_11_)(1′,2′-C_2_B_9_H_11_)], Cs[Me-COSAN]^28^; Cs[3,3′-Co(8-I-1,2-C_2_B_9_H_11_)_2_], Cs[I_2_-COSAN]^28^; and three compounds containing two COSAN moieties: Na_2_[1″,4″-{3,3′-Co(8-O(CH_2_CH_2_O)_2_-1,2-C_2_B_9_H_10_)(1′,2′-C_2_B_9_H_11_)}_2_-C_2_H_4_] (sodium ethylene glycol-diCOSAN)^29^; Na_2_[1″,4″-{3,3′-Co(8-O(CH_2_CH_2_O)_2_-1,2-C_2_B_9_H_10_)(1′,2′-C_2_B_9_H_11_)}_2_-C_6_H_4_] (sodium dihydroxybenzene-diCOSAN)^30^ and Na_2_[3,3′-Co(8-C_4_H_8_O_2_-1,2-C_2_B_9_H_10_)(1′,2′-C_2_B_9_H_11_)] (sodium dimethoxybenzene-diCOSAN)^31^ were synthesized as described in the literature. The water-insoluble compounds were dissolved in a mixture of acetonitrile/water (50:50) and passed repeatedly through a cation exchanging resin, which had been loaded previously with the desired cation (H^+^, Li^+^ or Na^+^). The solvent was removed by evaporation^29^. For cell experiments, all compounds were dissolved in KK2 (16.5 mM KH_2_PO_4_, 3.8 mM K_2_HPO_4_, pH 6.2) or phosphate-buffered saline (PBS) prior to addition to cell medium.

### Cell culture

Bacteria, *Dictyostelium* and mammalian cells were grown using standard laboratory procedures, appropriate for the cell type, e.g. Medium type, (LB-broth, HL5 medium or DMEM respectively), cell density and growth temperature. Cell numbers for maintain standard culture densities and measuring growth rates were measured by methods appropriate to cell type: optical density at 600 nm (OD_600_) for bacteria; direct cell counting with a haemocytometer for *Dictyostelium* cells; a combination of direct cell counting and luminescent detection of ATP (Cell-Titer-Glo®) for mammalian cells. Cells were visually detected an IX71 inverted DIC microscope, 40× objective (Olympus) and images captured using a Hamamatsu Orca ER camera. To calculate ED_50_, independent data sets were fit to an exponential curve, and individual ED_50_ values were calculated by interpolation. The mean (±SD) values of three independent ED_50_ values were calculated.

### Micro Raman spectroscopy

Samples were visualized with a Raman microscope system (Eclipse Ti-U, Nikon), stimulated with a diode pumped solid-state (DPSS) laser, analysed with a spectrometer (iHR550, Horiba) and acquired via a spectroscopy CCD camera (Newton, Andor). COSAN and I_2_-COSAN uptakes was determined for HEK293 cells in phosphate-buffered saline (PBS) by performing Raman single-point spectroscopy. Single cell Raman spectra were acquired from 20 cells^13^.

### Partition Coefficient (*P*) measurement

The Partition Coefficient (*P*) is defined as the ratio of the amount of compound present in the organic phase (*n*-octanol) to the amount present in the aqueous phase. Different amounts of Na[COSAN] (2.0, 2.4 and 3.6 mg) and Na[I_2_-COSAN] (0.9, 1.2 and 1.8 mg) have been added to a 10 ml vial containing 3 ml of *n*-octanol and 3 ml of deionized water. The different vials have been horizontally and vigorously shacked at room temperature for 2 hours at 350 rpm, to ensure the compound's transfer between the two phases. After, the vials are left standing for 1 hour in order to separate the phases, and later centrifuged 10 minutes at 6000 rpm. Finally, the organic and the aqueous phases have been transferred to a UV Cell and the UV absorption has been measured in a Shimadzu UV-Vis 1700 spectrophotometer, at the maximum absorbance of λ: 299 and 281 nm for Na[COSAN], in *n*-octanol and water, respectively; and 289 and 281 nm for Na[I_2_-COSAN], again in *n*-octanol and water, respectively. The interpolation of the obtained values in the previously prepared quantification curves allowed knowing the concentration of metallacarborane in each phase. Due to the partial solubility of *n*-octanol in water 0.032 g/100 g at 25°C, and of water in *n*-octanol 3.8 g/100 g, the calibration curves have been prepared with water saturated *n*-octanol and *n*-octanol saturated water, in order to avoid any possible medium interference.

## Author Contributions

M.T., C.V., F.T. and A.H. conceived the experiments, designed the project and wrote the paper. M.T., J.F., K.A., E.P. and E.C. performed the experiments.

## Supplementary Material

Supplementary Information Supplementary figure legends

## Figures and Tables

**Table 1:** Effective dose (ED_50_) for COSAN and I_2_-COSAN) on cell growth

	Compound ED_50_ (mean ± SD, μM)	
Cell type	COSAN	I_2_-COSAN
**Mammalian cells**		
HEK293	121 ± 3.8	30 ± 3.8
HeLa	157 ± 8.6	23 ± 2.5
THP-1	154 ± 8.9	44 ± 6.0
3T3	99 ± 5.5	29 ± 0.8
*Dictyostelium*	2.6 ± 0.3	1.8 ± 0.1
Bacteria		
*E. coli* B/r	nc^1^	53 ± 5.4
*Klebsiella pneumonia*	nc^1^	65 ± 4.1

^1^not calculated: unable to measure as ED_50_ is greater than 250 μM, above the solubility of COSAN in LB medium.
