# Supplementary material for: Biological interaction of living cells with COSAN-based synthetic vesicles
Source: Sci Rep. 2015 Jan 15;5:7804. doi: 10.1038/srep07804 (PMC4295085; doi:10.1038/srep07804)

Supplementary Figure legends:

Biological interaction of living cells with COSAN-based synthetic vesicles

Màrius Tarrés, Elisabetta Canetta, Eleanor Paul, Jordan Forbes, Karima Azzouni, Clara Viñas, Francesc Teixidor and Adrian J. Harwood

Supplementary figure legends

Figure S1: Measuring COSAN and I_2-COSAN.

(A) Figure shows calibration Raman spectra at different concentrations of I2-COSAN. The absorption at 2570 cm^{-1} (B-H vibration) is detectable above 10 mM, but not at 2 mM. Full spectra measured within cells incubated for 1 hour with 25 mM COSAN (B) and I_2-COSAN (C).

Fig1SA and 1SB are reproduced from Ref. 13 (http://pubs.rsc.org/en/content/articlehtml/2014/cc/c3cc49658a) by permission of The Royal Society of Chemistry.

Figure S2. Lipophilicity measurements studies by "shake-flask" n-octanol/water.

Calibration curves for: a) Na[COSAN] in *n*-octanol (λ_{max} = 299 nm); b) Na[I_2-COSAN] in *n*-octanol (λ_{max} = 289 nm); c) Na[COSAN] in H_2O (λ_{max} = 281 nm); d) Na[I_2-COSAN] in H_2O (λ_{max} = 310 nm). Vials after the shaking process containing: e) Na[COSAN] and f) Na[I_2-COSAN], mostly in the *n*-octanol phase.

Figure S3: Analysis of potential degradation of COSAN and I_2-COSAN degradation.

MALDI-TOF analysis of COSAN (A) and I_2-COSAN (B) extracted from *Dictyostelium* cell after 4 days of incubation. In both cases a single peak of molecular weight corresponding to the original of the starting compound. Predicted theoretical MS peak is shown below main figure.

Figure S1

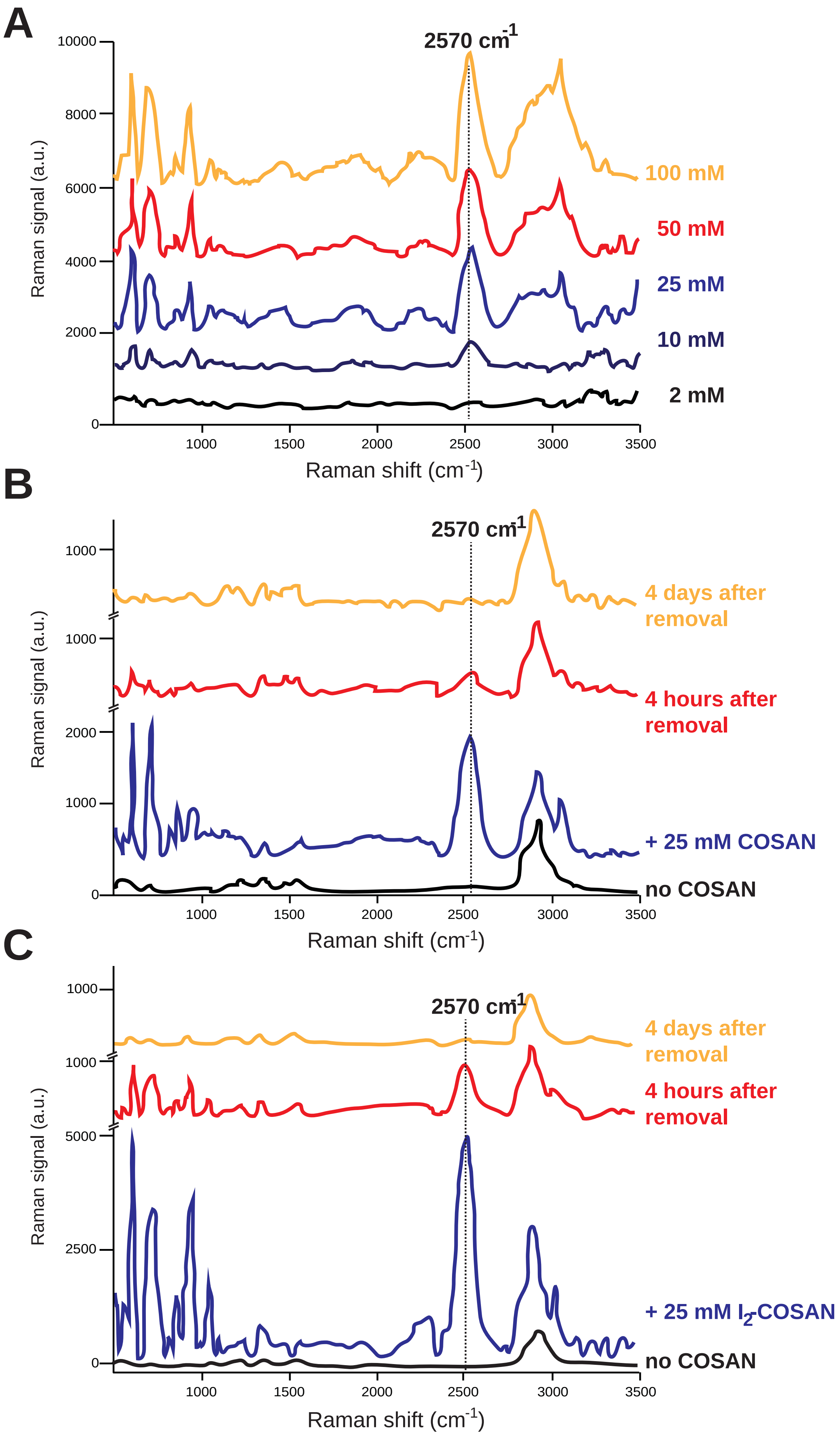
A
2570 cm-1
10000
8000
6000
4000
2000
0
Raman signal (a.u.)
100 mM
50 mM
25 mM
10 mM
2 mM
1000
1500
2000
2500
3000
3500
Raman shift (cm-1)
B
2570 cm-1
1000
1000
2000
1000
0
Raman signal (a.u.)
4 days after removal
4 hours after removal
+ 25 mM COSAN
no COSAN
Raman shift (cm-1)
C
2570 cm-1
1000
1000
5000
2500
0
Raman signal (a.u.)
4 days after removal
4 hours after removal
+ 25 mM I2-COSAN
no COSAN
Raman shift (cm-1)

Figure S2: Lipophilicity measurements studies by “shake-flask” n-octanol/water

a)

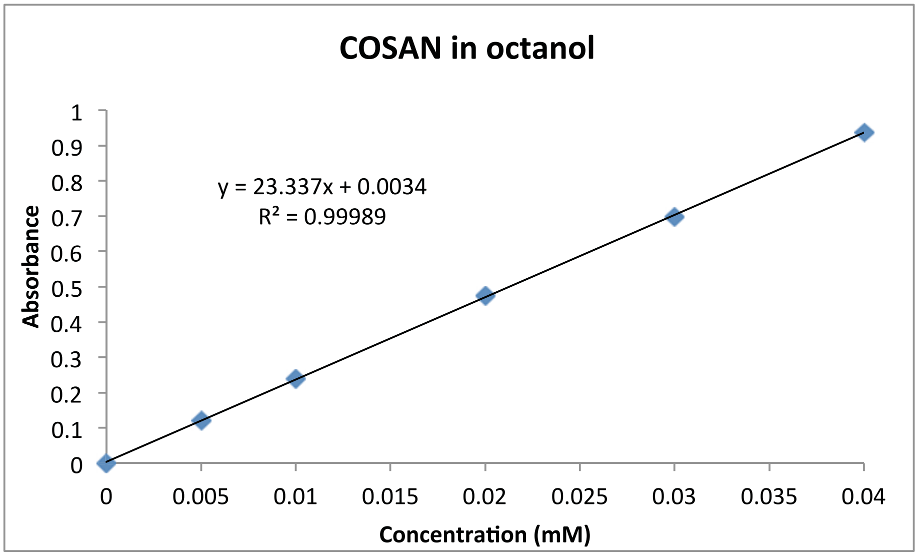

b)

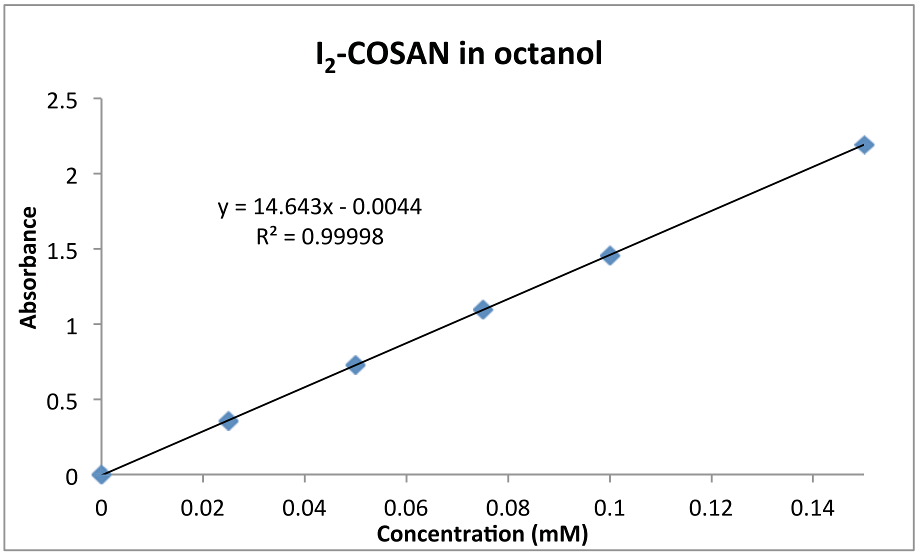

c)

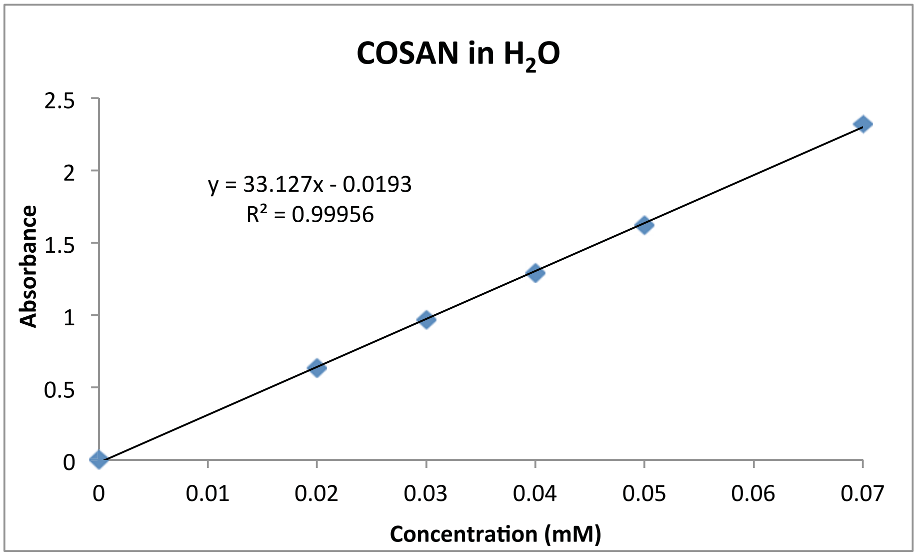

COSAN in H_2O
y = 33.127x - 0.0193
R^2 = 0.99956
Absorbance
2.5
2
1.5
1
0.5
0
0
0.01
0.02
0.03
0.04
0.05
0.06
0.07
Concentration (mM)

d)

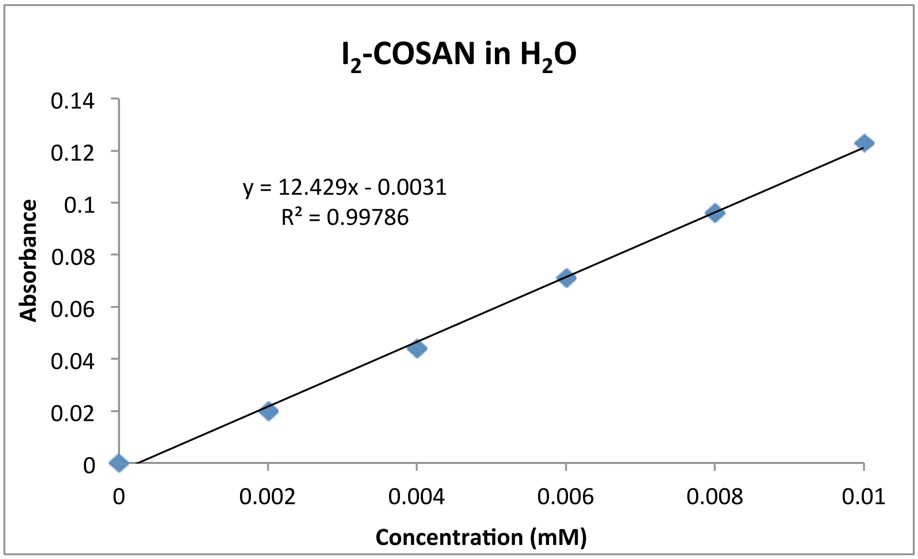

I_2-COSAN in H_2O
y = 12.429x - 0.0031
R^2 = 0.99786
Absorbance
0.14
0.12
0.1
0.08
0.06
0.04
0.02
0
0
0.002
0.004
0.006
0.008
0.01
Concentration (mM)

e)

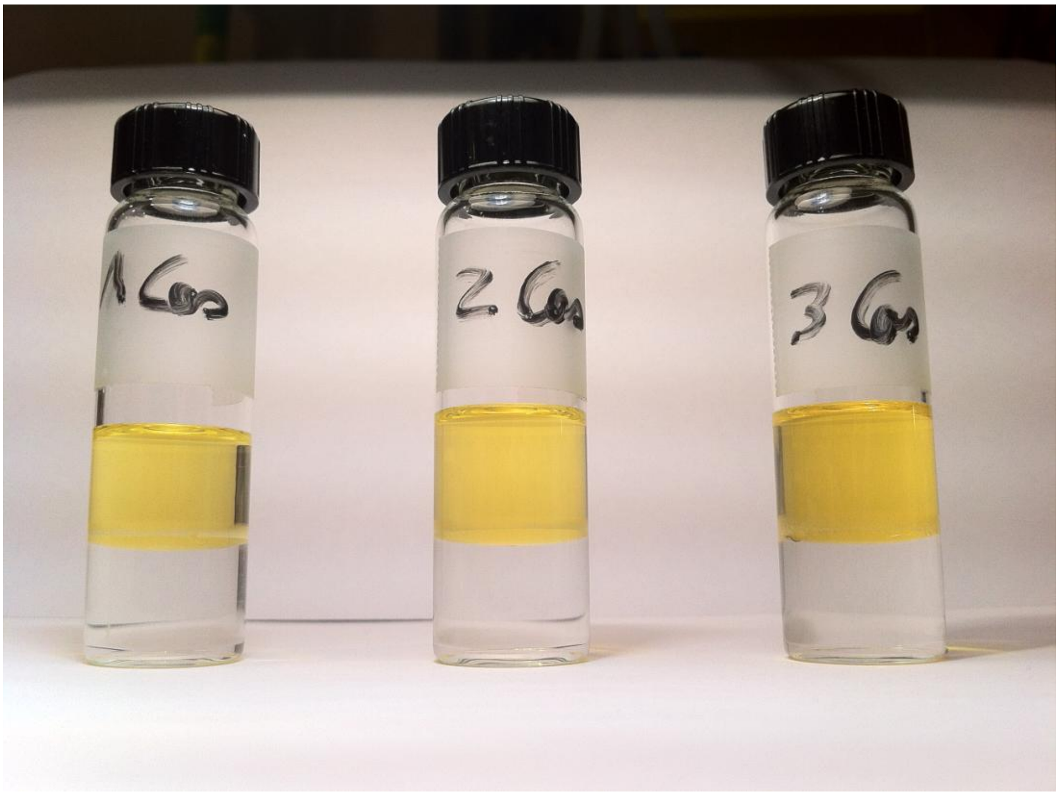

f)

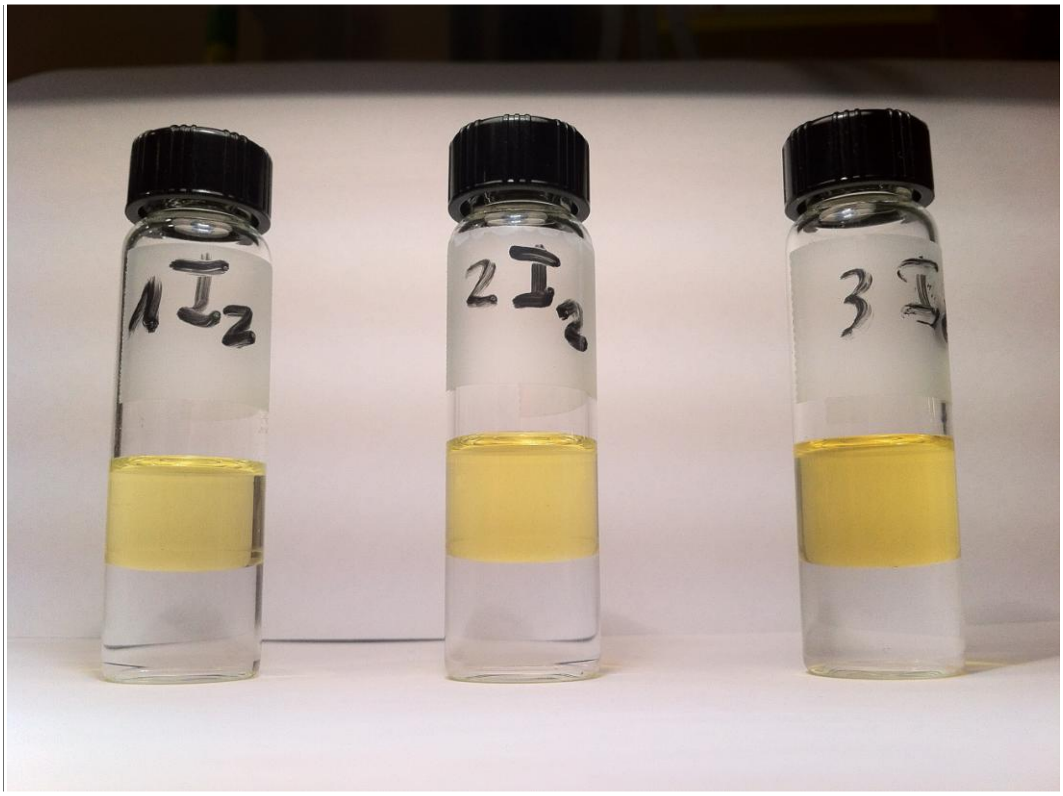

Figure S3

A: MALDI-TOF MS of COSAN. a) Full experimental spectrum; b) Magnification of the experimental spectrum; c) Magnification of the theoretical spectrum.

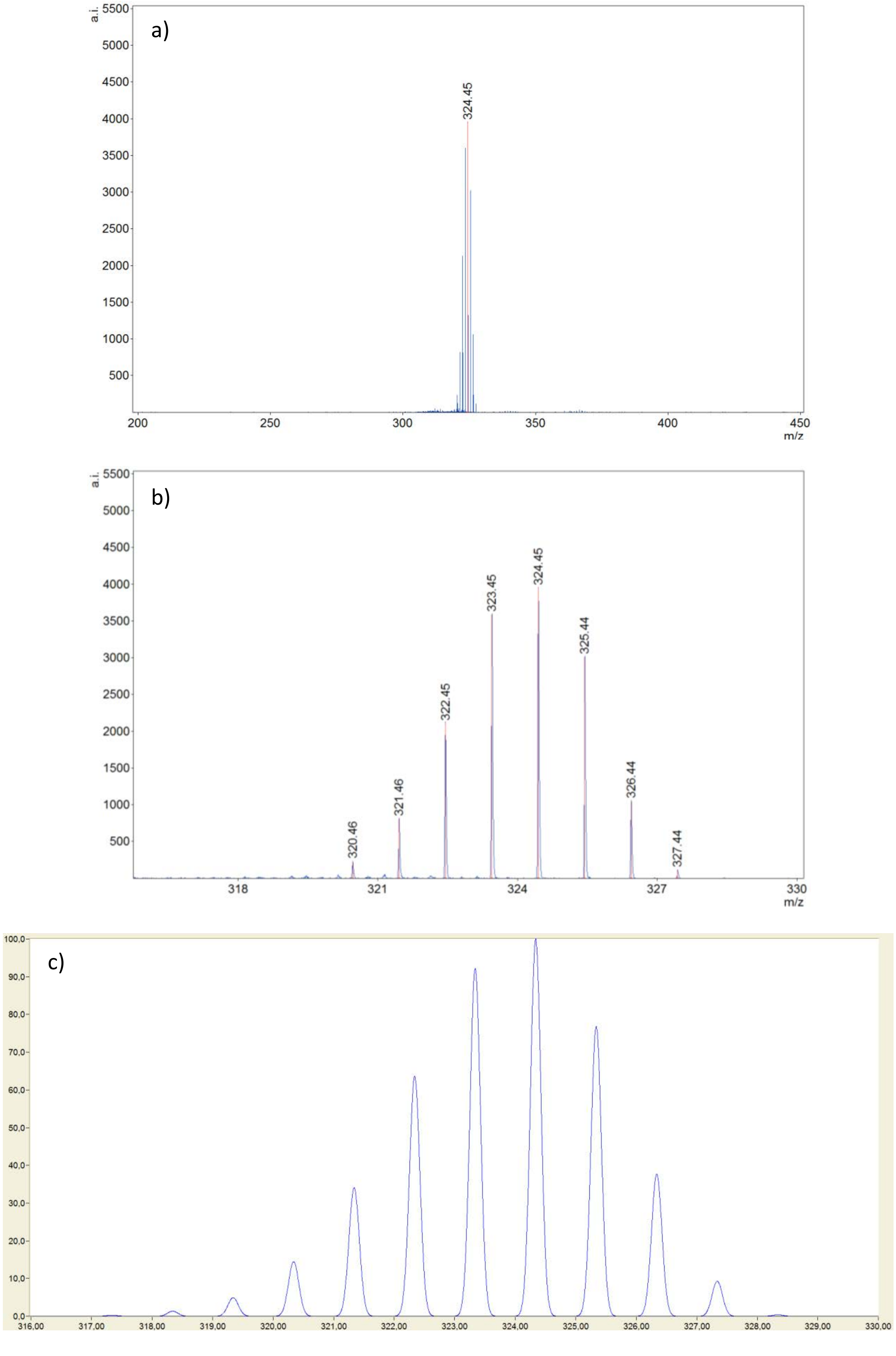

B: MALDI-TOF MS of I_2-COSAN. a) Full experimental spectrum; b) Magnification of the experimental spectrum; c) Magnification of the theoretical spectrum.

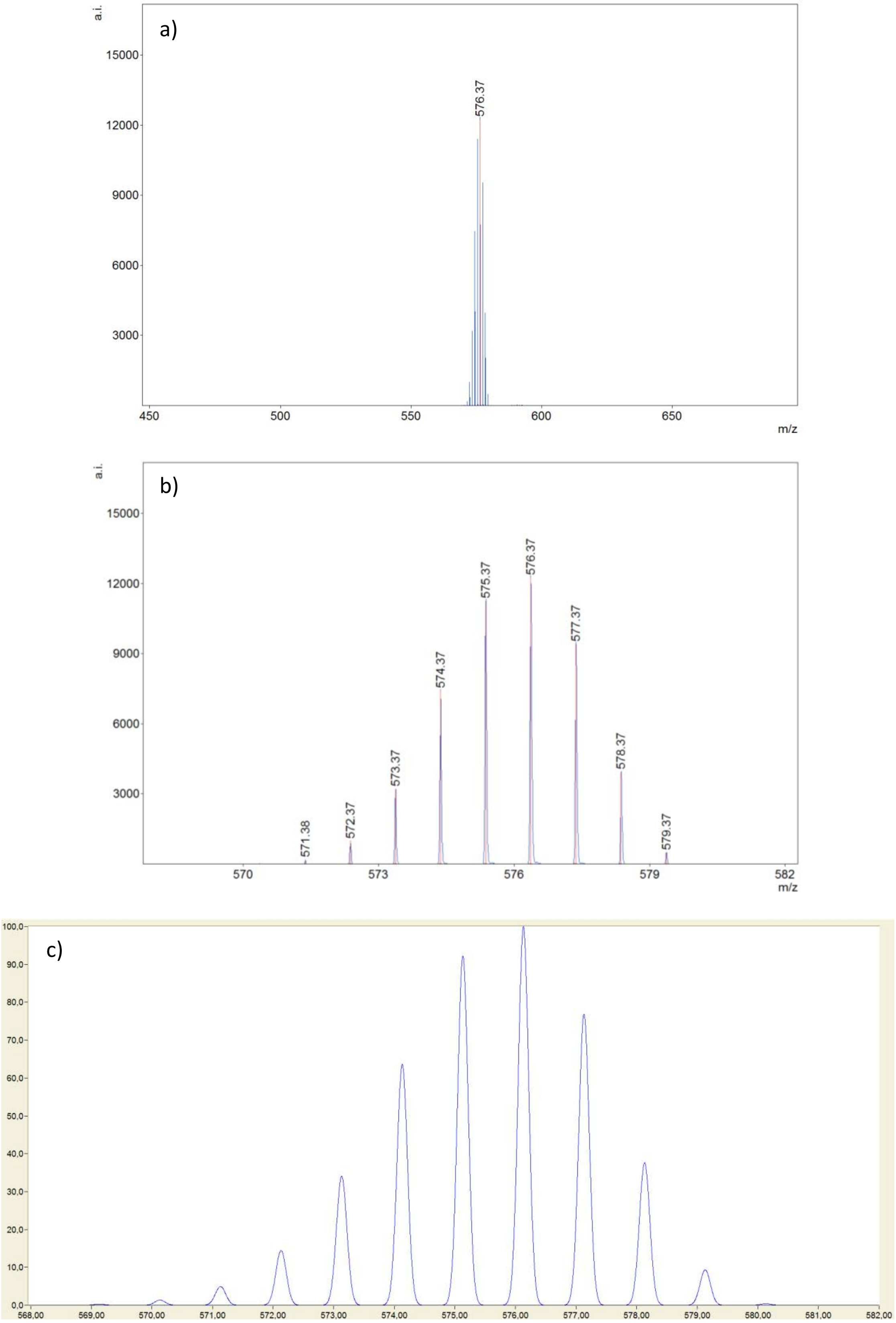

Supplement: Supplementary Information — Supplementary figure legends [file srep07804-s1.pdf]
